# Development of a Neighbourhood Walkability Index for Porto Metropolitan Area. How Strongly Is Walkability Associated with Walking for Transport?

**DOI:** 10.3390/ijerph15122767

**Published:** 2018-12-06

**Authors:** Ana Isabel Ribeiro, Elaine Hoffimann

**Affiliations:** 1 EPIUnit—Instituto de Saúde Pública, Universidade do Porto, 4050-600 Porto, Portugal; elainehoffimann@gmail.com; 2 Department of Public Health, Forensic Sciences and Medical Education, University of Porto Medical School, 4200-319 Porto, Portugal

**Keywords:** built environment, urban health, urban form, walking, physical activity, health promotion

## Abstract

The creation of walkable communities constitutes a cost-effective health promotion strategy, as walking is an accessible and free intervention for increasing physical activity and health. In this cross-sectional ecological study, we developed a walkability index for the Porto Metropolitan Area and we validated it by assessing its association with walking for transportation. Neighborhood walkability was measured using a geographic information system and resulted from the weighted sum of residential density, street connectivity, and a destination-based entropy index. The index was categorized into quintiles of increasing walkability. Among the 1,112,555 individuals living in the study area, 28.1% resided in neighborhoods in the upper quintile of walkability and 15.8% resided in the least walkable neighborhoods. Adjusted regression models revealed that individuals residing in the most walkable neighborhoods are 81% more likely to report walking for transportation, compared with those from the least walkable neighborhoods (odds ratio: 1.81; 95% confidence intervals: 1.76–1.87). These results suggest that community design strategies to improve walkability may promote walking behavior.

## 1. Introduction

Despite the many benefits of physical activity (PA) [1], most people do not engage in regular PA. The Portuguese remain amongst the most physically inactive Europeans, as 68% of Portuguese adults were reported as never engaging in PA [2].

Walking is the most common form of PA. It is affordable, enjoyable, and versatile; it is recognized as a means of increasing levels of PA for the majority of the population [3]. Furthermore, walking can be easily incorporated into daily life routines, namely during trips from home to work/school, so-called “walking for transportation” (or active transportation).

Certain built environmental characteristics can act as facilitators or as barriers to walking. For instance, a well-connected street network shortens distances between origins and destinations, and the availability of a variety of destinations (recreation, services, and retail) within a walkable distance from residence reduces automobile trips and promotes walking [4].

To combine key built environmental features that encourage walking behavior—street network connectivity, land-use mix, and residential density—the walkability index was developed in the 2000s [5]. An increasing number of studies, mainly from the United States (US) and Australia, showed that neighborhood walkability is an important correlate of PA, i.e., walking behavior [3,6,7], suggesting that the creation of more walkable communities may constitute a powerful and cost-effective tool for promoting PA at population-level.

Despite the importance of addressing the built environmental correlates of PA, no validated measures of neighborhood walkability exist in Portugal. The walkability index was mostly applied in North America and Australia. Given the differences in urban form, extrapolation of findings to European cities such as Porto may not be appropriate [8]. Cities in America are more decentralized and sprawling, while cities in Europe are more central and compact [9]. Furthermore, the walkability index proposed by Frank and colleagues uses a set of well-established variables that were shown to be significantly associated with walking behavior [5]. Ideally, the same set of variables should be used in Porto (Portugal) to create a similar index. However, these variables were not available and alternate indexes must be computed. The creation of novel and modified walkability indexes requires validation to assess if they preserve the same explanatory power of the original index. In 2016, two indexes of walkability were developed for the Porto municipality [10,11], though these indexes were not validated by measuring their association with walking behavior. This is particularly relevant because the previously mentioned indexes used land-use data from a pan-European dataset—European Environment Agency Urban Atlas land-use map [12]—which has poor attribute accuracy. For instance, commercial, industrial, and military land-uses are collapsed in the same land-use category (“industrial, commercial, public, military, and private units”), which may not allow for the accurate quantification of the diversity of non-residential destinations, a key component of the walkability index.

Taking into account these research gaps, we aimed to develop a neighborhood walkability index for the Porto Metropolitan Area and to test if it is associated with walking for transportation.

## 2. Materials and Methods

### 2.1. Study Area

The Porto Metropolitan Area is the second largest metropolitan area of Portugal. It is located in the northern region of the country and has a population of 1.3 million [13].

For the development of the walkability index, we focused on the six core municipalities of the Metropolitan Area, which hold roughly 85% of its population (*N* = 1,112,555 inhabitants): Porto, Matosinhos, Maia, Vila Nova de Gaia, Gondomar, and Valongo. These municipalities are divided into 10,444 census tracts. Census tracts are an operational unit for data collection and constitute the smallest geographical unit of census data dissemination (mean of 107 inhabitants/area, mean area of 53,852 m^2^) [14]. Census tracts, from here onward simply referred to as neighborhoods, were used as the unit of analysis in the present study.

### 2.2. Walkability Index

Neighborhood walkability is generally composed of four elements [6]. Due to data unavailability, the ratio of retail building floor areas was not included; thus, our index incorporated the remaining three variables: residential density, street connectivity, and entropy index. Similarly to us, due to data unavailability, several other studies were also forced to remove this variable [7,8,10,15,16]. Despite this methodological modification, these studies were able to detect a significant association between the developed walkability index and walking for transport [7,8,15,16], which indicates that the explanatory power of the index may not be affected by this omission.

All the variables and procedures required to assess the components of neighborhood walkability are described in Table 1.

#### 2.2.1. Residential Density

Residential density was obtained by calculating the density of households (number of households per km^2^) within each neighborhood. For that, data from the 2011 population and housing census, available at Statistics Portugal (https://www.ine.pt/), were used.

#### 2.2.2. Street Connectivity

An updated street network dataset, provided courtesy of Environmental Systems Research Institute (ESRI), was used to compute the number of intersections (≥3 intersecting streets) per km^2^ within 800 m of the centroid of each neighborhood; only streets that allowed pedestrian circulation were included. For this assessment, we used the ArcGIS 10.4 Network Analyst tool (Environmental Systems Research Institute, ESRI, Redlands, CA, USA) and an 800-m (equivalent to a 10-min walk) threshold as employed in other studies focused on measuring pedestrian access to neighborhood facilities [17,18]. Additionally, to evaluate how sensitive our results were to the distance threshold, the 400-m distance was also employed. The 800-m and 400-m buffers contain approximately 2,011,000 and 503,000 square meters, respectively.

#### 2.2.3. Entropy Index

To assess the variety of destinations available to each neighborhood, we computed an entropy index. Traditionally, the entropy index is based on the percentage area occupied by different types of land uses that promote PA, and reflects how varied the neighborhood is in terms of its land-use distribution [5,6]. However, no accurate polygon dataset of those land uses was openly available for the Porto Metropolitan Area. Thus, we computed an alternative, destination-based measure of entropy as described below.

(1) Step 1: Collection of the locations of commonly visited destinations in the study area. Based on previous studies [6,19], we considered the following types/groupings of destinations: retail, institutional, services, recreational, and residential. Several sources of data were used, fully detailed in Table 1. These data sources can be considered reliable and accurate, as details about most destinations were centralized in institutional and territorial local authority websites and datasets.
(2) Step 2: Assessment of the number of destinations of each type within 800 m of the centroid of each neighborhood, using the ArcGIS 10.4 Network Analyst tool and an updated street network. Again, for sensitivity analysis, the 400-m distance threshold was also employed.
(3) Step 3: After determining the number of destinations of each type at a walkable distance, we calculated the entropy index for each neighborhood, using the equation below.
  (1) $$Entropy\;index\;of\;j\;neighbourhood=\frac{-\sum p_{ij}\times ln\;p_{ij}}{lnN_{i}},$$
  where $p_{ij}$ is the fraction of destinations of type *i* at a walkable distance from neighbourhood centroid *j*, and $N_{i}$ is the number of different destination types, i.e., five. Values may range from 0 (low diversity of destinations) to 1 (high diversity).

#### 2.2.4. Walkability Index Calculation

Each of the previously described variables was standardized, and the walkability score was obtained by summing the three z-scores [10].

The walkability index was created to be used both as a continuous score and/or a categorical variable. As in the original version of the measure [5], the neighborhood walkability index was then categorized in quintiles of increasing value (Q1—least walkable to Q5—most walkable).

### 2.3. Walking for Transport

Data on the number of individuals that report walking from/to school/work were obtained from the 2011 population and housing census, available via Statistics Portugal (https://www.ine.pt/).

In the census survey, every inhabitant answered the following question: What is your main mode to travel to school/work? (1, walking; 2, car; 3, bus; 4, collective transport offered by school/work; 5, metro; 6, train; 7, motorcycle; 8, bike; 9, boat; 10, other). The person could select one option only. There were no missing data, as usual in universal population censuses.

In this study, we used the number of individuals (count) living in each neighborhood who reported walking to/from home/work and, as a denominator, we included the total number of individuals living in the same neighborhood.

### 2.4. Covariates

We also included data on potential confounders in the association between neighborhood walkability and walking for transport, identified in previous studies [5,20]: the population age and gender distribution (proportion of active-age population 15–64 years old, and proportion of women), the proportion of employed individuals, and the proportion of people working in other municipalities. These data were obtained from the 2011 population and housing census, available via Statistics Portugal (https://www.ine.pt/), Additionally, we considered the neighborhood socioeconomic deprivation index developed and fully described in previous publications [21,22]. In short, this index was calculated from the weighted sum of the following standardized variables: % overcrowded households, % households with no bath/shower, % households without indoor flushing, % households occupied by non-owners, % women aged 65 or more, % individuals with low education, % individuals in low-income occupation, and % unemployed individuals. The index was categorized into quintiles of increasing socioeconomic deprivation (Q1—least deprived to Q5—most deprived).

### 2.5. Associations with Transport Walking

Generalized additive models (GAM) were used to estimate the association between the proportion of individuals that reported walking from/to school/work and the walkability index score and quintiles.

GAM extends generalized linear models to include nonparametric smoothing. This approach, employed previously [23,24], allowed us to model the spatial distribution of the response variable, thus allowing for the control of spatial autocorrelation.

Firstly, we fitted a univariable model, which included the response variable, the walkability index, and a function (thin plate spline) applied on the coordinates of each neighborhood.

Then, the model was adjusted for potential confounders: the population age and gender distribution (proportion of active-age population 15–64 years, and proportion of women), the proportion of employed individuals, the proportion of people working in other municipalities, and the neighborhood socioeconomic deprivation index. Statistical analyses were conducted in the R software version 3.3.3. (R Foundation for Statistical Computing, Vienna, Austria) using the package “mgcv”.

## 3. Results

**Figure 1:**
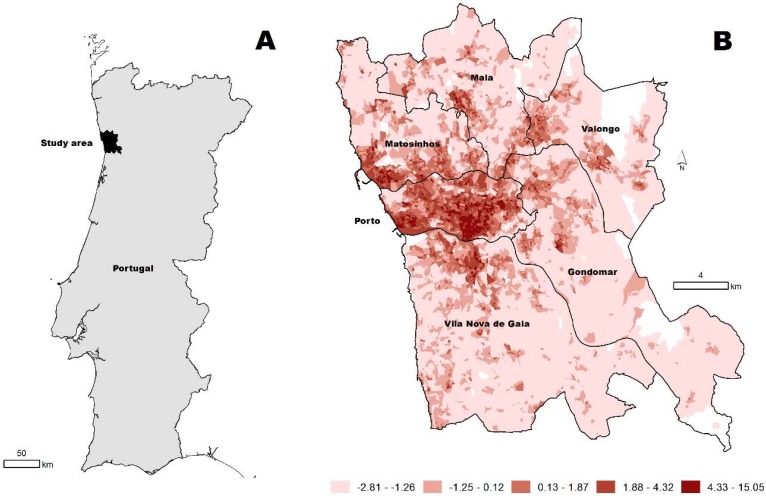
Location of the study area in Portugal (**A**). Spatial distribution of the walkability index at the neighborhood level (*n* = 10,444) (**B**).

The spatial distribution of the walkability index is shown in Figure 1. The average walkability index in the study area was 0.01, ranging from −2.81 to 15.05. Out of the 1,112,555 inhabitants living in the study area, 15.8% resided in neighborhoods in the first quintile of walkability (Q1—least walkable), 16.0% in Q2, 17.9% in Q3, 22.2% in Q4, and 28.1% in Q5 (the most walkable neighborhoods). For the geographical distribution of the index, we observed a clear radial decay in the walkability index of the Porto Metropolitan Area from the center (Porto municipality) to the periphery (municipalities of Matosinhos, Maia, Valongo, Gondomar, and Vila Nova de Gaia) (Figure 1).

Table 2 shows the population characteristics. Overall, 15.4% of the population reported walking to/from work/school. Table 3 depicts the association between the walkability index and the proportion of the population that walks to/from work/school. There is a graded and positive association between the walkability index and transport-related walking.

This association remained after adjustment for potential confounding variables. Compared with the least walkable neighborhoods, individuals residing in the most walkable neighborhoods were significantly more likely to walk to/from work/school (Q1 vs. Q5 odds ratio: 1.81; 95% confidence intervals: 1.76–1.87). When using the walkability index score, we observed that the odds of walking to/from work/school increased by 7% for every unit increase in the walkability index (odds ratio: 1.07; 95% confidence intervals: 1.07–1.08).

Similar results were obtained using the 400-m distance threshold, as shown in Table S1 (Supplementary Materials).

## 4. Discussion

The present study was designed to measure of how well the Porto Metropolitan Area neighborhoods promote active forms of transportation, such as walking. This study fulfilled an important evidence gap in Portugal, where, despite the extremely low levels of PA, few investments were made in ascertaining how friendly urban environments are for pedestrian movement. To address this, we developed a walkability index at the neighborhood level by adapting an original methodological framework for walkability [5] to the data available in the study area.

Confirming findings from international investigations [3,6,7,8,15,16,25,26], we observed a positive and graded association between neighborhood walkability and walking. Residents in more walkable neighborhoods—areas with a variety of destinations within walking distance, a well-connected street network, and a high density of residences—presented 81% higher odds of walking for transportation (to/from work/school), as compared with those living in the least walkable areas. While our methods are not fully comparable with all those used in other studies on the topic, it is important to note that the effect sizes observed in the literature somehow match ours. For instance, Chudyk et al. reported that a 10-point increase in the walkability index score was associated with 45% greater odds of walking for transportation in a population of older adults from Canada [3]. In a sample of South Asians living in the US, for each 10-point increase in walk score, men engaged in 13 additional minutes per week of walking for transport [16]. Villanueva et al. collected data from a sample of Australian adults, and observed that a point increase in the walkability index score was associated with 6% greater odds of walking [7]. In Sweden, individuals living in highly walkable neighborhoods, compared to those living in less walkable neighborhoods, had 77% higher odds of walking for active transportation [15]. Studies using objective measures of walking, derived from accelerometry, also observed a positive association between neighborhood walkability and daily steps [25,26].

Regarding, the geographical distribution of the index, we observed a clear radial decay in the walkability index of the Porto Metropolitan Area from the center (Porto municipality) to the periphery, as expected. This is likely the case because the Porto municipality is the urban core, and the peripheral municipalities (Matosinhos, Valongo, Maia, Vila Nova de Gaia, and Gondomar) have lower land-use mix and street connectivity, two of the walkability index components. Similar radial decay patterns in the walkability index were observed in other urban areas [6,8,27].

This investigation has a number of limitations that should be discussed. Concerning the index development methodology, we could not include all possible destinations available in the study area (namely shops and small food outlets), due to difficulty in accessing high-quality and accurate datasets. This may affect the absolute walkability index value by underestimating it, but it is unlikely to influence the relative position of the neighborhoods, i.e., the quintiles. We were also unable to obtain information on a fourth variable commonly included (ratio of retail building floor areas). Concerning the measurement of associations, conclusions regarding causality and the directionality of the associations could not be made, given the study’s cross-sectional design. We relied on a dichotomous (yes/no) self-reported variable to measure walking for transport, which might lead to recall and reporting bias. Previous studies found a low-to-moderate correlation between self-reported and objective measures of PA [28]. Thus, ideally, objective measures obtained using global position systems (GPS) and accelerometry, should be used to measure walking for transport [28]. Additionally, our variable on walking for transport did not capture the duration and intensity of walking, which are more relevant to health.

This study also has a number of strengths and implications. Firstly, we developed, for the first time, a validated walkability index for Porto area, which is critical for establishing priorities for the creation of more walkable and pedestrian-friendly communities. Secondly, we developed and tested an alternative measure of entropy, which may be useful in settings where no high-resolution land parcel maps are openly available. The methods employed in this study may be easily transposed and applied in other countries of high, medium, and low income, where accurate and updated land-use datasets may not be available, provided these new indexes are subject to proper validation. Moreover, we tested the impact of using different thresholds of geographical proximity to destinations (800 and 400 m); this is critical as there is no current consensual definition of what a walkable distance is. In sum, the validation of the index was based on census data, which have universal coverage in Portugal, and included data from 1.1 million citizens living in the second largest metro area of the country.

Demonstrating that the way the urban environment is built influences walking behavior provides important evidence for the development of area-based interventions for the creation of more PA-friendly communities. Moreover, the creation of walkable communities may lead to a series of co-benefits [29]: (1) significant health gains at population level, as physical inactivity is a leading risk factor for mortality and several non-communicable diseases [30]; (2) environmental sustainability, since it reduces car trips, which minimizes air pollution and noise [29]; (3) economic gains, by decreasing expenses related to car ownership and strengthening the local business environment [29].

## 5. Conclusions

In conclusion, this study showed that neighborhood walkability has a positive and graded association with walking for transportation. Although we cannot directly infer this from our study, our results suggest that implementing community design strategies to improve walkability might enhance PA among Portuguese urbanites, specifically by increasing walking for transportation. The study also demonstrated that, in the absence of land-use maps, an alternative and valid measure of the neighborhood entropy index could be developed based on the number and type of destinations within a certain area.

## Figures and Tables

**Table 1:** Data sources and procedures used for assessing the characteristics of the built and socioeconomic environment of the neighborhoods.

Variable		Data Source	GIS and Statistical Procedure
Residential density		2011 Census available at Statistics Portugal (https://www.ine.pt/).	We computed the ratio of the dwellings per neighborhood area.
Street connectivity		ESRI StreetMap for ArcPad Portugal TomTom (http://enterprise.arcgis.com/en/streetmap-premium/latest/get-started/dd-tomtom-data.htm).	Firstly, we removed the intersections of 2 streets or less, as well as intersections of motorways. Then, we computed the density of intersections (intersections per km^2^) within 400 and 800 m of the neighborhood centroid.
Entropyindex	Retail	Shopping centers, markets, and supermarkets obtained in 2018 from online business directories.	Whenever needed, destinations were georeferenced using Google Maps and ArcGIS Online Geocoding Service. Most destinations had a location represented by a single point; however, for green spaces, we used the entrances of these spaces. Then, using the ArcGIS Network Analysist tool, we determined the number of destinations of each type within 400 and 800 m of the neighborhood centroid. Finally, the index was obtained using the entropy index equation.
	Recreation	Restaurants, sport facilities, green spaces, libraries, zoos, art galleries, and museums obtained in 2018 from the TLA databases and online business directories.	
	Services	Banks, post-offices, pharmacies, hospitals, primary care centers, finance office, credit unions, courts, and notary, obtained in 2018 from the TLA databases, institutional websites, and online business directories.	
	Institutional	Schools, universities, kindergartens, churches, city halls, police stations, and fire stations, obtained in 2018 from the TLA databases, institutional websites, and online business directories.	
	Residential	Number of exclusively residential buildings obtained from the 2011 census available at Statistics Portugal (https://www.ine.pt/).	

ESRI—Environmental Systems Research Institute; TLA—territorial local authorities.

**Table 2:** Characteristics of the population residing in the study area (*N* = 1,112,555).

Variables	%
Gender (men)	47.4
Active-age population 15–64 years	68.5
Employed individuals	60.3
Working in other municipalities	24.1
Walking from/to work/school	15.4
Neighborhood walkability index	
Q1—least walkable	15.8
Q2	16.0
Q3	17.9
Q4	22.2
Q5—most walkable	28.2
Neighborhood socioeconomic deprivation	
Q1—least deprived	15.5
Q2	19.1
Q3	22.4
Q4	20.9
Q5—most deprived	22.2

**Table 3:** Crude and adjusted associations between the neighborhood walkability index and the proportion of residents walking from/to work/school.

	Walking from/to Work/School OR and 95% CIs ^1^	Walking from/to Work/School AOR and 95% CIs ^2^
Neighborhood walkability index		
Q1—least walkable	1.00	1.00
Q2	1.08 (1.05–1.11)	1.11 (1.08–1.15)
Q3	1.30 (1.27–1.34)	1.37 (1.33–1.41)
Q4	1.44 (1.40–1.48)	1.56 (1.51–1.60)
Q5—most walkable	1.53 (1.48–1.57)	1.81 (1.76–1.87)
Neighborhood walkability index (score)	1.05 (1.04–1.05)	1.07 (1.07–1.08)

^1^ Odds ratio (OR) and 95% confidence intervals. ^2^ Adjusted odds ratio (AOR) and 95% confidence intervals. Adjusted for the proportion of active-age population 15–64 years, proportion of men, proportion of employed people, proportion of people working in other municipalities, and neighborhood socioeconomic deprivation score.

## Supplementary Materials

The following are available online at http://www.mdpi.com/1660-4601/15/12/2767/s1: Table S1: Crude and adjusted associations between the neighborhood walkability index and the proportion of residents walking from/to work/school (threshold distance equal to 400 m).
