# Supplementary material for: Development of a Neighbourhood Walkability Index for Porto Metropolitan Area. How Strongly Is Walkability Associated with Walking for Transport?
Source: Int J Environ Res Public Health. 2018 Dec 6;15(12):2767. doi: 10.3390/ijerph15122767 (PMC6313616; doi:10.3390/ijerph15122767)
Supplement: Supplementary file 1 [file ijerph-15-02767-s001.pdf]

Table S1. Crude and adjusted associations between the neighbourhood walkability index and the proportion of residents walking from/to work/school (threshold distance equal to 400 m).

	**Walking from/to Work/School OR and 95% CI [1]**	**Walking from/to Work/School AOR and 95% CI [2]**
Neighbourhood walkability index		
Q1—least walkable	1.00	1.00
Q2	1.13 (1.09–1.16)	1.14 (1.11–1.18)
Q3	1.26 (1.23–1.30)	1.30 (1.26–1.34)
Q4	1.40 (1.37–1.44)	1.47 (1.43–1.52)
Q5—most walkable	1.59 (1.55–1.64)	1.72 (1.67–1.77)

[1] Odds ratio (OR) and 95% Confidence Intervals. [2] Adjusted Odds ratio (AOR) and 95% Confidence Intervals. Adjusted for the proportion of active-age population, 15–64 years, proportion of men, proportion of employed people, the proportion of people working in other municipalities and the neighbourhood socioeconomic deprivation score.
